# Targeting Cardiovascular Risk Factors Through Dietary Adaptations and Caloric Restriction Mimetics

**DOI:** 10.3389/fnut.2021.758058

**Published:** 2021-09-30

**Authors:** Julia Voglhuber, Senka Ljubojevic-Holzer, Mahmoud Abdellatif, Simon Sedej

**Affiliations:** ^1^Department of Cardiology, Medical University of Graz, Graz, Austria; ^2^BioTechMed Graz, Graz, Austria; ^3^Centre de Recherche des Cordeliers, Equipe labellisée par La Ligue Contre le Cancer, Université de Paris, Sorbonne Université, INSERM U1138, Institute Universitaire de France, Paris, France; ^4^Faculty of Medicine, Institute of Physiology, University of Maribor, Maribor, Slovenia

**Keywords:** cardiovascular risk factors, obesity, hypertension, caloric restriction mimetics, autophagy, dietary regimens, caloric restriction, intermittent fasting

## Abstract

The average human life expectancy continues to rise globally and so does the prevalence and absolute burden of cardiovascular disease. Dietary restriction promotes longevity and improves various cardiovascular risk factors, including hypertension, obesity, diabetes mellitus, and metabolic syndrome. However, low adherence to caloric restriction renders this stringent dietary intervention challenging to adopt as a standard practice for cardiovascular disease prevention. Hence, alternative eating patterns and strategies that recapitulate the salutary benefits of caloric restriction are under intense investigation. Here, we first provide an overview of alternative interventions, including intermittent fasting, alternate-day fasting and the Mediterranean diet, along with their cardiometabolic effects in animal models and humans. We then present emerging pharmacological alternatives, including spermidine, NAD^+^ precursors, resveratrol, and metformin, as promising caloric restriction mimetics, and briefly touch on the mechanisms underpinning their cardiometabolic and health-promoting effects. We conclude that implementation of feasible dietary approaches holds the promise to attenuate the burden of cardiovascular disease and facilitate healthy aging in humans.

## Introduction – A brief Overview of Cardiovascular Risk Factors

Cardiovascular diseases remain the major cause of morbidity and mortality, accounting for 17.9 million deaths per year or almost one third of all deaths worldwide^1^,^1^. Functional decline of the cardiovascular system and increased vulnerability to disease manifestation is accelerated by various risk factors. While some risk factors, such as age, sex, family history and race are unmodifiable, several behavioral and environmental risk factors can be efficiently targeted through lifestyle modifications and/or pharmacological interventions. In this regard, systemic analyses of global cardiovascular disease trends and patterns revealed a cluster of modifiable cardiovascular risk factors, including high blood pressure, obesity, diabetes mellitus type 2, and hyperlipidemia, which are on the rise due to the global population aging, hypercaloric dietary habits, and sedentary lifestyle (1).

Extensive body of evidence indicates that hypertension is the leading modifiable risk factor for cardiovascular disease and premature mortality (2), accounting for 9.4 million global deaths per year (3). In 2010, around 31.1% of adult population worldwide (or 1.39 billion) were reportedly hypertensive (4). As such, high blood pressure remains an unmet medical need, despite the widespread use of antihypertensive medications. Obesity and unhealthy diets are major behavioral determinants that hamper the long-term control of hypertension (2), contributing to the increased risk for cardiovascular disease (5). Furthermore, obesity-related high levels of low-density lipoprotein-cholesterol (LDL-cholesterol) and triglycerides are causally linked to the progression of atherosclerosis (6), a chronic inflammatory disease associated with increased risk of ischemic cardiomyopathy and myocardial infarctions. Diabetes mellitus type 2 is a global health risk that is often seen concurrently with obesity and obesity-related complications, resulting in a 2-fold increase of cardiovascular disease risk (7). Despite accumulating evidence of the detrimental role of obesity and diabetes mellitus type 2 in the development of cardiovascular disease, their prevalence has reached epidemic dimensions^2^. Importantly, the increasing co-occurrence of multiple morbidities, such as obesity, dyslipidemia, diabetes mellitus type 2, and hypertension, which are referred to as a cluster of conditions also known as metabolic syndrome, typically contributes to an exponential increase in the risk for cardiovascular diseases (8).

In general, patients with cardiovascular disease are commonly affected by more than one risk factor (9). Emerging evidence suggests that most cardiovascular diseases can be prevented using systematic approaches that target behavioral risk factors such as unhealthy diet, obesity, and physical inactivity. Indeed, dietary restrictions or regular exercise have recently attracted much attention for cardiovascular disease prevention as recent estimations suggest that preventive treatments might reduce the development of cardiovascular disease by 80% (3). However, most patients exhibit low adherence to such demanding lifestyle modifications. Therefore, there is a pressing need to identify alternative interventions with better compliance. Various natural and pharmacological supplements or small molecules have emerged as potential candidates to replicate the pleiotropic salutary effects of dietary restriction and, thus, might offer better adherence without reducing calorie intake.

**Figure 1:**
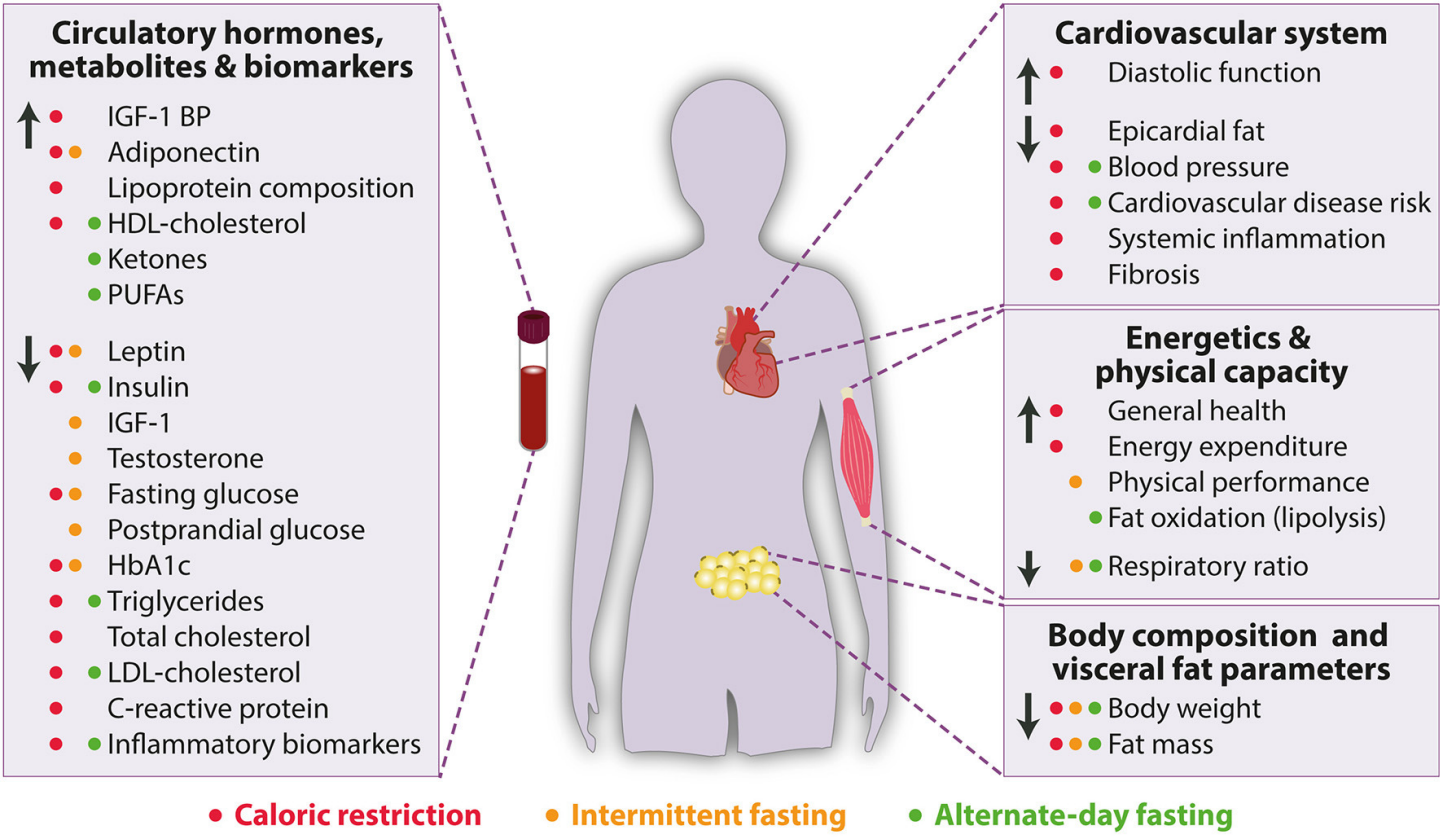
Beneficial effects of caloric restriction (red), intermittent fasting (orange) and alternate-day fasting (green) on cardiometabolic parameters in humans. HbA1c, Glycated hemoglobin; HDL, High-density lipoprotein; IGF-1, Insulin-like growth factor-1, IGF-1 BP, Insulin-like growth factor-1 binding protein; LDL, Low-density lipoprotein; PUFAs, Polyunsaturated fatty acids. Arrow up indicates increase or improvement, arrow down indicates decrease or decline.

The amount of dietary intake, quality of food and its preparation as well as micronutrient composition together with general eating habits (e.g., meal timing and frequency) significantly contribute to the onset of cardiovascular disease risk factors (3, 10). To this end, many studies have tested various forms of dietary modifications for their efficiency on improving cardiovascular and metabolic health (Table 1 and Figure 1).

**Table 1:** Overview of human trials testing the efficacy of dietary interventions on cardiometabolic risk.

**Dietary intervention**	**Diet characteristics and duration**	**Follow-up time**	**Disease/target population**	**Study design/Number of participants/Sex**	**Effect**	**Study outcomes**	**Reference/Trial title**
Mediterranean diet	Until follow-up	4.8 years	High-risk for cardiovascular disease	RCT 7,447 participants 57% women	↓	30% reduced cardiovascular disease risk	(11)
	12 weeks Therapeutic lifestyle changes (diet plus exercise)	-	Metabolic syndrome	Prospective pilot study 25 participants 76% women	↓ ↑	Body weightBody-mass-indexFasting insulinHDL function	(12)
	1-year intervention Enriched with extra-virgin olive oil or nuts	-	High-risk for cardiovascular disease	RCT 296 participants 51% women	↑	HDL atheroprotective functions	(13) PREDIMED
	1-year intervention Enriched with extra-virgin olive oil or nuts	-	High-risk for cardiovascular disease	RCT 1,139 participants 55% women	↓ ↑	LDL-cholesterolInflammatory biomarkers (VCAM-1, intracellular adhesion molecule, IL-6, TNFα, monocyte chemotactic protein 1)HDL-cholesterol	(14) PREDIMED
	3-month intervention Enriched with extra-virgin olive oil or nuts	-	High-risk for cardiovascular disease	RCT 49 participants 53% women	↓	Pro-atherothrombotic genes	(15) PREDIMED
	Enriched with extra-virgin olive oil or nuts	4.1 years	High-risk for cardiovascular disease	RCT 3541 participants 70% women	↓	Diabetes risk	(16) PREDIMED
	Increased fish consumption (non-fried)	10 years	Healthy post-menopausal women	Observational study 84,493 women	↓	Heart failure risk	(17) WHI-OS
	During early adulthood increased fish (non-fried) and long chain omega-3 PUFAs	25 years	Young adults, free form metabolic syndrome and diabetes	Prospective cohort study 4,356 participants 53% women	↓	Metabolic syndrome incidence	(18) CARDIA
Caloric restriction (CR)	2-year intervention 25% CR	-	Healthy, non-obese	RCT 220 participants 67% women	↓↑	Body weightGeneral health	(19) CALERIE 2
	2-year intervention 25% CR	-	Healthy, non-obese	RCT 53 participants (analyzed) 68% women	↓	10-year cardiovascular disease risk by 30%Blood pressureBody weightSubcutaneous and visceral fatInsulin resistance (at 12 months of intervention)LDL-cholesterolCholesterolTriglycerides	(20) CALERIE 2
	6-month intervention 25% CR plus other groups with exercise and varied % of CR	-	Overweight	RCT 48 participants 57% women	↓	Body weightFat massLeptin	(21) CALERIE
	2-year intervention 25% CR	-	Normal weight to moderately overweight	RCT 218 participants 68% women	↓ ↑	Body weightBlood pressureInsulin resistanceInflammatory biomarkers (triiodothyronine, TNFα)TriglyceridesLDL-cholesterolTotal cholesterolEnergy expenditureHDL-cholesterol	(22) CALERIE
	6-month intervention	-	Metabolic syndrome	Observational study 18 men	↓ ↑	Body weightInsulin levelsFasting glucosePro-inflammatory cytokinesLipoprotein composition	(23)
	6-month intervention 25% CR, additional subgroup for 2 days/week	-	Healthy, obese or overweight, family history of breast cancer in 54% of participants	RCT 107 women	↓ ↑	Body weightBlood pressureFasting insulinInsulin resistanceLeptinC-reactiveProteinLDL-cholesterolTriglyceridesIGF-1 BP	(24)
	16-week intervention Calorie reduction of 700 or 500 kcal/day (latter coupled to physical exercise)	-	Diabetes mellitus type 2	RCT 63 participants 51% women	↓ =	Body weightEpicardial fatTotal fat massCardiometabolomic profile	(25)
	20-week intervention calorie deficit of ~400 kcal/day	-	Older, heart failure with preserved ejection fraction	RCT 92 participants 80% women	↑	Peak oxygen consumption	(26)
	CR for 6.5 ± 4.6 years	-	Healthy	Cross-sectional 50 participants 19% women	↓ ↑	Blood pressure C-reactive protein TNFα, TGFβ_1_Diastolic function	(27)
Intermittent fasting	2-week intervention ~17 h fasting cycles	-	Diabetes mellitus type 2 + metformin, obese	Observational study 10 participants 90% women	↓ ↑	Body weightMorning glucose levelPostprandial glucose levelPhysical activity	(28)
	8-week intervention 16 h fasting cycles	-	Healthy men	RCT 34 men	↓ ↑	Fat massIGF-1TestosteroneRespiratory ratioAdiponectin	(29)
Alternate-day fasting	8-week intervention Allowed for 25% of energy intake on fasting days	-	Obese	Interventional study 16 participants 75% women	↓	Body weightBody fat percentageBlood pressureTotal LDLLDL-cholesterolTriglycerols	(30)
	22-day intervention No control group	-	Non-obese	16 participants 50% women	↓ ↑	Body weightFasting insulinRespiratory quotientFat oxidation	(31)
	4-week and 6-month intervention	-	Healthy non-obese	Cohort study with integrated pilot RCT 90 participants for long term ADF 58% women57 participants in RCT 60% women	↓ ↑	Cardiovascular disease riskFat-to-lean ratioInflammatory markers (sICAM-1, triiodothyronine) LDL-cholesterolKetones PUFAs	(32) InterFast
	8-week intervention High-fat (45%) or low-fat (25%) diet on non-fasting days	-	Obese	RCT 32 women	↓	Coronary heart disease riskBody weightFat massLDL-cholesterolTriacylglycerol	(33)
Intermittent fasting *vs*. caloric restriction	12-week intervention Continuous CR (5,000–6,500 kJ/day) or intermittent fasting for 2 days/week	-	Overweight/obese and Diabetes mellitus type 2	RCT 63 participants 52% women	↓	Body weight HbA1cComparable results between IF and CR	(34)
Alternate-day fasting *vs*. caloric restriction	1-year intervention 25% of energy intake allowed on fasting days or 25% CR continuously	-	Obese	RCT 100 participants 86% women	↓ ↑	Body weightHDL-cholesterol in alternate-day fasting (6 months of intervention)	(35)
	3-week intervention 150 or 200% energy intake on non-fasting days or 25% CR continuously	-	Healthy and lean	RCT 36 participants 58% women	↓ ↑	Body weight (not for 200% energy intake)Body fat (not for 200% energy intake)LDL-cholesterol (only CR)LeptinHDL-cholesterolAdiponectinCR more effectively reduces body weight than alternate-day fasting with 25% reduced energy intake, which confers no additional short-term metabolic or cardiovascular benefits	(36)

*We searched the US clinical trial registry (https://www.clinicaltrials.gov/) and PubMed using terms “Mediterranean diet,” “Caloric restriction,” “Intermittent Fasting,” “Alternate-day fasting,” and “Cardiovascular risk/disease” for completed, pending or ongoing clinical trials testing the effects of dietary regimes on cardiovascular risk factors*.

*HDL, High-density lipoprotein; IGF-1, Insulin-like growth factor-1; IGF-1 BP, Insulin-like growth factor-1 binding protein; IL-6, Interleukin-6; LDL, Low-density lipoprotein; PUFA, Polyunsaturated fatty acid; RCT, Randomized clinical trial; sICAM-1, Soluble intercellular adhesion molecule-1; TGFβ_1_, Transforming growth factor β_1_; TNFα, Tumor necrosis factor α; VCAM-1, Vascular cell adhesion protein-1*.

*↑ (arrow up) indicates increase or improvement, ↓ (arrow down) indicates decrease or decline, = indicates no change*.

## Dietary Approaches for Improving Cardiometabolic Health

### Mediterranean Diet

The Mediterranean diet is characterized by high fruit and vegetable intake combined with plenty of fish and unsaturated fatty acids derived mainly from extra-virgin olive oil, with minimal or no consumption of low saturated fat and processed food. Many epidemiological studies and randomized clinical trials report that the traditional Mediterranean diet is associated with lower risk for all-cause and cardiovascular disease mortality, coronary heart disease, metabolic syndrome, and diabetes mellitus type 2 (37, 38). For example, a meta-analysis demonstrated 10% reduction in cardiovascular disease incidence or mortality, and 8% decrease in all-cause mortality (39). In similar vein, a randomized controlled trial (PREDIMED) that included high-risk individuals consuming the Mediterranean diet showed that the cardiovascular disease risk could be lowered by almost 30% (11). In a sub-study derived from the PREDIMED principal trial, the Mediterranean diet was found to improve high-density lipoprotein (HDL) atheroprotective functions (13). Remarkably, similar effects on HDL function were reported in individuals suffering from metabolic syndrome, which were subjected to the Mediterranean diet coupled to exercise for 12 weeks only (12). Increased polyphenol intake from Mediterranean diet is associated with improved levels of LDL-cholesterol, HDL-cholesterol, and systolic and diastolic blood pressures in older participants at high risk for cardiovascular disease. Furthermore, elevated polyphenol consumption reduces circulating inflammatory biomarkers, such as vascular cell adhesion protein-1, interleukin-6, tumor necrosis factor-α, which are linked to atherosclerosis (14). Another sub-study of the PREDIMED trial reported reduced expression of genes involved in vascular inflammation, foam cell formation and thrombosis in a high cardiovascular disease risk population (15).

Growing evidence suggests that the markedly reduced risk for cardiovascular disease by the Mediterranean diet is attributed to its plant-rich nutrient composition with seafood as the predominant source of animal protein. For example, the Women's Health Initiative Observational Study demonstrated that increased consumption of baked or boiled fish, but not fried fish, inversely correlates with heart failure risk in postmenopausal women (17). In agreement with these findings, a 25-year follow-up study suggested that increased intake of long-chain omega-3 polyunsaturated fatty acids (PUFAs) and non-fried fish in early adulthood protects against the development of metabolic syndrome (18). Similarly, the Mediterranean diet enriched with extra-virgin olive oil, but without reduced caloric intake, reduces the risk for diabetes mellitus type 2 in individuals with high cardiovascular risk (16). In sum, the Mediterranean diet is a promising and feasible diet with manifold cardiometabolic benefits. However, since the PREDIMED trial has had its limitations (40), additional randomized clinical studies are warranted to corroborate the efficacy of this most extensively studied dietary regimen.

### Caloric Restriction

Caloric restriction and other forms of stringent eating behaviors, such as intermittent fasting and alternate-day fasting, have recently attracted a lot of attention amongst researchers and have become increasingly popular in the general population to avoid the unhealthy effects of “all-around-the-clock” high caloric diet. Caloric restriction is defined as a chronic reduction of overall calorie consumption without malnutrition. In patients with metabolic syndrome, caloric restriction reduces body weight and exerts beneficial effects on insulin levels, fasting glucose levels, lipoprotein composition and pro-inflammatory cytokines within 6 months of intervention (23). In addition to weight loss in obese or overweight women, caloric restriction reduces leptin, total C-reactive protein (CRP), LDL-cholesterol, triglycerides, blood pressure, fasting insulin and insulin resistance (24). In another study, improved body weight and reduced epicardial fat accumulation were also observed in patients with diabetes mellitus type 2 subjected to caloric restriction. These effects were further augmented by physical activity, while cardiometabolic profiles were apparently unchanged (25).

Caloric restriction was shown to be a safe and well-tolerable intervention in healthy, non-obese individuals (41), leading to body weight loss (19, 20, 42), reduced fat mass and waist circumference (20, 21, 42), and improved general health (19). A long-term clinical trial reported increased energy expenditure without negatively affecting the quality of life in non-obese to moderately overweight cohorts (22). A 20-week long intervention with caloric restriction also potently improved peak oxygen consumption in older and obese patients with heart failure with preserved ejection fraction (26). Beneficial effects of caloric restriction were attributed to reduced blood pressure (20, 22, 24, 42), and lower total cholesterol and LDL-cholesterol concentrations (20) as well as lower leptin levels (21), altogether contributing to reduced 10-year risk for cardiovascular disease by 30% (20). Caloric restriction exerts cardiac-specific effects that ameliorate aging-related decline in diastolic function (27). These salutary effects on heart function might be mediated by the effect of caloric restriction on blood pressure, systemic inflammation, and cardiac fibrosis (43).

Mechanistically, the beneficial effects of caloric restriction are closely linked to autophagy, a cellular recycling process essential for cardiovascular homeostasis (44, 45). Caloric restriction mediates positive effects on the heart also via increased activity of SIRT1 and peroxisome proliferator-activated receptor gamma coactivator 1-α (PGC1α), leading to reduced amount of reactive oxygen species (ROS), and less fibrosis and inflammation (46). Furthermore, caloric restriction lowers oxidative stress in the heart and vasculature by increasing the expression of endothelial nitric oxide synthase (eNOS), and activating superoxide dismutase (SOD) and NADPH oxidase (47). Importantly, non-cell autonomous mechanisms also contribute to the cardiovascular health benefits of prolonged caloric restriction. Although the mechanisms are still ill-defined, the “metabolic switch” hypothesis may explain, at least in part, improvements in cardiovascular health indicators, such as lower blood pressure in animals and humans (48). In fact, fasting induces the conversion of hepatic fatty acids into ketone bodies (e.g., β-hydroxybutyrate), which act as fuel and potent signaling molecules, with the capacity to effectively reduce markers of inflammation and control various regulators of systemic metabolism, such as levels of HDL and LDL cholesterol, triglycerides, and glucose (48, 49).

Notably, severe caloric restriction (~800 kcal per day) induces changes of gut microbiome composition during weight loss (50, 51). However, the consequences of gut microbiome composition alteration for health and disease in response to stringent caloric restriction are only beginning to unveil. A very recent clinical trial, with 80 post-menopausal women who were overweight or obese, revealed that severe calorie restriction imparts a reversible shift in the gut microbiome associated with improved glucose regulation and decreased adiposity, indicating improved metabolic health in dieters (52).

Collectively, caloric restriction exerts clear cardiometabolic benefits in both obese and non-obese individuals. However, caloric restriction might also cause adverse side effects on immunity, fertility and bone density. Hence, further research is warranted to develop more suitable dietary patterns or pharmacological alternatives to reproduce the health benefits of caloric restriction.

### Intermittent and Alternate-Day Fasting

In an effort to circumvent the complexity of counting calories and avoid the side effects associated with caloric restriction, other forms of dietary restriction with food intake limited to a daily time window, such as intermittent fasting and alternate-day fasting, have been proposed. Accordingly, different lengths of eating and fasting periods have been tested, with the most common reported of 16/8 h of fasting and eating intervals, respectively (53). Longer fasting periods of 24 h followed by *ad-libitum* food intake for 24 h are also practiced and known as alternate-day fasting. Although intermittent fasting and alternate-day fasting are not as well-studied as caloric restriction, emerging evidence suggests that they are more tolerable and their side effects are less prominent than in caloric restriction and, thus, both dietary interventions could represent promising and more feasible strategies to curtail the hypercaloric pandemic in the Western societies (54).

To this end, a study comparing the efficacy of caloric restriction and intermittent fasting (restricted to 2 days a week) in obese diabetic patients at risk of cardiovascular disease showed that both regimens reduce body weight and HbA1c levels, a measure of long-term blood glucose control (34). Consistently, another small observational study on obese subjects with diabetes mellitus type 2 and receiving metformin reported that short-term intermittent fasting effectively reduces body weight and improves morning glucose levels. Interestingly, 6 out of 10 participants in this study described that intermittent fasting is highly tolerable, and reported readiness to follow intermittent fasting after study completion (28). Of note, intermittent fasting was capable to improve health parameters in healthy, male athletes. Specifically, intermittent fasting reduced body fat mass without worsening body fat-free mass, muscle area and strength. These effects were associated with lower concentrations of insulin-like growth factor-1 (IGF-1) and higher adiponectin levels, while leptin was not found reduced after adjusting for body fat mass (29). By contrast, a recent meta-analysis concluded that the evidence supporting a positive effect of intermittent fasting on glucose remains uncertain, despite the robust body weight-lowering effect (55). Interestingly, the analysis suggested that both intermittent fasting and caloric restriction equally improve cardiometabolic risk factors. Irrespectively, larger studies with long-term follow-up are necessary to clearly determine the effect of either regimen on hard cardiovascular end-points, such as myocardial infarction, heart failure as well as cardiac and all-cause mortality.

With regard to alternate-day fasting, a short-term trial conducted in obese adults, which showed high adherence to alternate-day fasting at least for 8 weeks, revealed manifold cardiometabolic benefits, including reduced body weight, body fat percentage, total and LDL-cholesterol, triglycerides as well as systolic blood pressure (30). It is important to mention that the participants were allowed for 25% energy intake on fasting days. Interestingly, short-term alternate-day fasting effectively reduces body weight, body fat mass and waist circumference despite high-fat dietary intake on non-fasting days. However, although alternate-day fasting improves plasma levels of LDL-cholesterol and triacylglycerol in obese individuals, HDL-cholesterol, blood pressure and heart rate are not altered (33). At variance with short-term studies, a long-term trial reported low adherence to the prescribed amount of energy intake and, accordingly, a high dropout of obese, otherwise metabolically healthy adults subjected to alternate-day fasting within the 1-year follow-up (35). This study also included a caloric restriction group, which exhibited higher compliance rates than the alternate-day fasting group. Although reduction in body weight was evident upon both alternate-day fasting and caloric restriction, none of the fasting regimens improved blood pressure, plasma lipid profile, or markers of glucose control and inflammation. In addition, HDL-cholesterol levels that were higher at 6 months of alternate-day fasting, were not improved after 12 months (35). Recently, a 3-week randomized trial, which is among the first to disentangle the effects of alternate-day fasting and “traditional” daily energy restriction, revealed that alternate-day fasting without energy restriction is not sufficient to reduce body weight in lean individuals. However, although alternate-day fasting with 25% reduced energy intake reduces body mass, the decrease of body fat content is lower compared to a matched traditional daily energy restriction and confers no additional short-term metabolic or cardiovascular benefits (36). Further studies with larger cohorts and longer duration are warranted to examine the fasting-specific effects of alternate-day fasting and intermittent fasting, and directly compare their effects to diets that only reduce daily net calories.

Along similar lines, initial short-term studies in non-obese individuals highlighted the positive impact of alternate-day fasting on body weight loss in absence of clear metabolic changes, but increased fat oxidation. Notably, participants reported difficulty to adhere to alternate-day fasting due to severe hunger on the fasting days (31). By contrast, the InterFast trial showed that alternate-day fasting is capable of improving cardiometabolic markers in healthy non-obese subjects, including reduced body weight, fat-to-lean ratio, and LDL-cholesterol (32). Furthermore, alternate-day fasting increases ketone bodies (on fasting and non-fasting days), and reduces the inflammatory marker sICAM-1, suggesting that alternate-day fasting is a viable dietary adaptation also for non-obese individuals. Importantly, this 4-week long intervention trial reported no adverse effects on immunity or bone density.

In sum, growing body of evidence indicates potential cardiovascular benefits of intermittent and alternate-day fasting (56). However, it is still not clear whether these nutritional regimens, wherein food intake is limited to a consistent time-restricted interval without changes in nutritional quality or quantity, confer a significantly better adherence than caloric restriction. Also, it remains elusive whether the cardiometabolic benefits of these regimens can be applied to the general healthy population or specific groups with disorders, such as obese individuals with metabolic disease. Hence, larger studies, preferably with long-term follow-up, will be required to address these open issues.

## Caloric Restriction Mimetics

**Figure 2:**
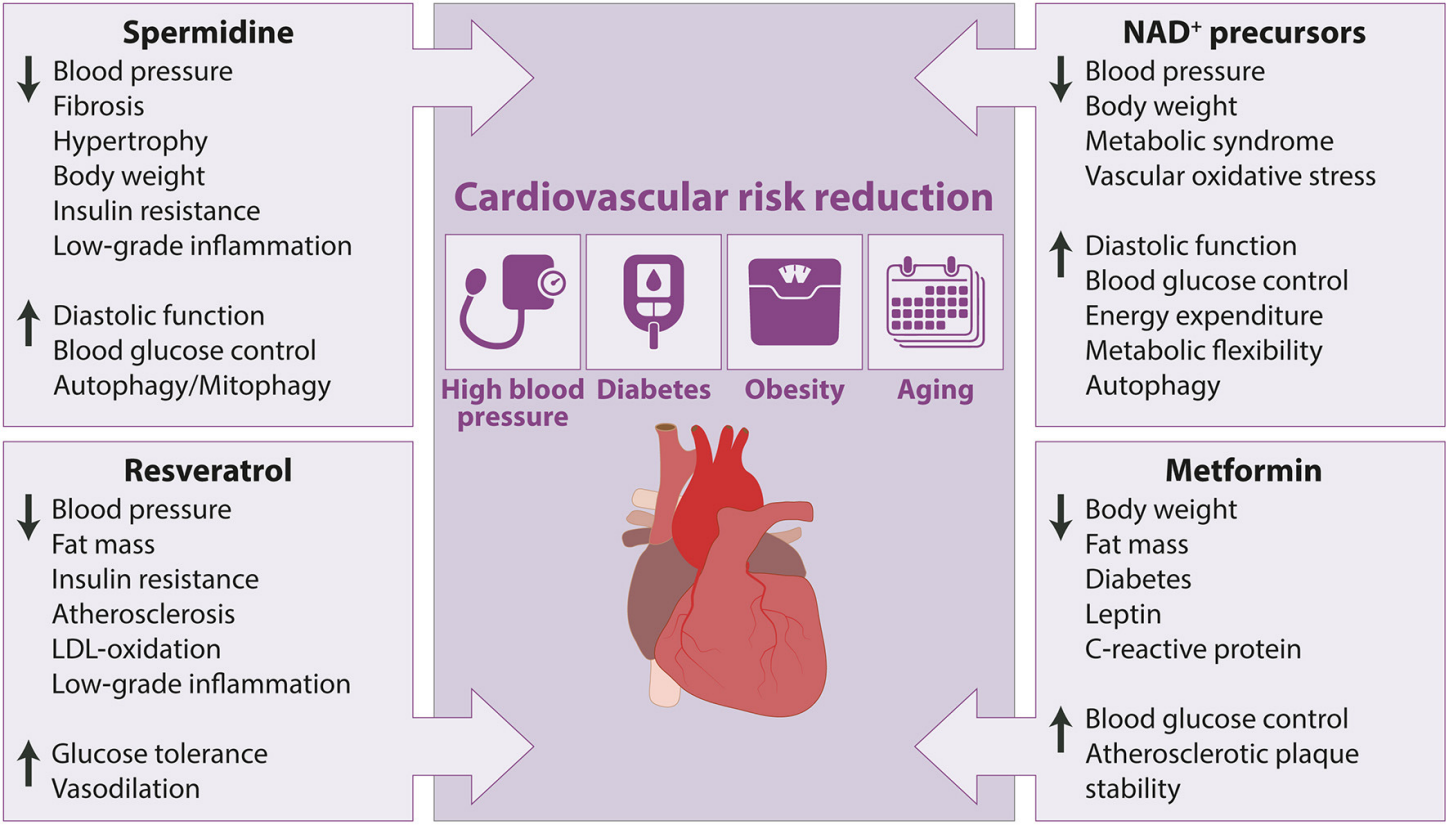
Cardiovascular and metabolic health-promoting effects of caloric restriction mimetics in animal models with cardiovascular risk factors. Arrow up indicates increase or improvement, arrow down indicates decrease or decline.

Recent years have seen an increasing interest in fasting-mimicking diets and caloric restriction, which might offer a more feasible alternative to stringent forms of fasting. For example, a randomized clinical trial was designed to investigate the effects of fasting mimicking diets, which are low in carbohydrates and protein and high in unsaturated fats, on cardiovascular disease and risk factors, including aging and diabetes mellitus type 2 (57). The authors observed that practicing low calorie fasting mimicking diet for only 5 consecutive days per month results in a reduction of body mass index (BMI), arterial blood pressure, fasting glucose, and IGF-1 levels. Generally, subjects who are at greater risk for disease, exhibit a larger benefit than individuals who have no other risk factors, confirming the relevance of fasting mimicking diet for disease prevention. Similarly, caloric restriction mimetics–natural and pharmaceutical compounds with intrinsic pro-autophagic action–might offer superior compliance, and are under intensive investigation as they have been shown to improve cardiovascular health and they might be used for the treatment of cardiovascular disease (58). Therefore, in the following section commonly used and well-studied caloric restriction mimetics will be discussed. Further, we will briefly describe their mode of actions and summarize the current evidence for the cardiovascular and metabolic effects of selected caloric restriction mimetics (Figure 2).

### Spermidine

Spermidine is a natural polyamine and autophagy inducer that exerts pleiotropic cardioprotective effects by lowering high blood pressure in salt-sensitive *Dahl* rats, while reducing maladaptive hypertrophy and attenuating the decline of diastolic function (59, 60), and arterial elastance in aged mice (61). In addition to its direct cardioprotective effects, accumulating evidence demonstrated the anti-obesity impact of spermidine supplementation in rodents consuming a high-fat diet (HFD). In particular, spermidine counteracts HFD-induced body weight gain and obesity-associated alterations by increasing lipolysis in visceral fat and improving blood glucose control in obese mice (62, 63), and diabetic rats (64). Interestingly, spermidine treatment appears to provide no additional metabolic benefit in young and old mice consuming normal chow (59, 63), suggesting that salutary metabolic effects of spermidine might be limited to hypercaloric and pro-diabetic dietary regimens. Beside the regulation of lipid metabolism, spermidine attenuates inflammatory response in the adipose tissue by decreasing inflammatory cytokine and chemokines expression (65). Spermidine is also capable of reducing circulating TNFα levels during aging, thereby counteracting chronic low-grade inflammation in old mice (59).

The cardiovascular health-promoting effects of spermidine supplementation are predominantly attributed to its cytoprotective autophagy-inducing properties. For example, cardiomyocyte-specific Atg5-deficient mice exhibit no cardiac benefits upon spermidine supplementation (59), while the aortic rings of spermidine-fed mice display no functional advantages over their non-treated controls upon incubation with the autophagy inhibitor chloroquine (61). Autophagy-inducing capacity of spermidine relies on the inhibition of several acetyltransferases, including EP300, one of the major negative regulators of autophagy (66). These autophagy-stimulatory properties are mediated via hypoacetylation of histones (67), and autophagy-related genes, such as *Atg5, Atg7*, and *Atg8* (68). In addition, spermidine has been proven to inhibit the mammalian target of rapamycin complex 1 (mTORC1) (66), a key regulator of cell growth and metabolism, and to activate AMP-dependent protein kinase (AMPK) (69). More recently, spermidine was reported to stimulate autophagy through the hypusination of eukaryotic translation initiation factor 5A-1 (eIF5A), which in turn controls the expression of transcription factor EB (TFEB), a master regulator of lysosome biogenesis and autophagy (70). By contrast, age-related decline of spermidine levels and subsequent down-regulation of TFEB may cause reduced autophagic activity in the adaptive immune system, as well as in other tissues. However, although many protective effects of spermidine are autophagy-dependent and associated also with increased mitophagy, a selective form of autophagy that degrades dysfunctional mitochondria (59, 71), a recent study showed that enhanced lipolysis by spermidine was independent of autophagy in adipose tissue (63). Indeed, spermidine effectively stimulated lipolysis in HFD-fed mice with adipose-specific autophagy deficiency. In this regard, further studies are warranted to elucidate, which of the cell type/tissue/organ-specific effects induced by spermidine requires autophagy.

In humans, circulating spermidine levels decline with age (72), and reduced endogenous concentrations of spermidine appear to be associated with age-related deterioration of cellular homeostasis attributed to decreased autophagy (73). The upregulation of endogenous spermidine levels extends lifespan across multiple species, including mice. Spermidine is abundantly found in wheat germ, soybeans, and nuts (73), and reportedly enriched also in the Mediterranean diet (74). While the optimal concentration of spermidine in humans to maintain optimal autophagy levels for healthy aging still needs to be determined, self-reported dietary spermidine intake has been shown to inversely correlate with arterial blood pressure, risk of both fatal and overt heart failure as also other cardiovascular disease (59), and overall mortality (75).

Taken together preclinical evidence supports the translational potential of spermidine to ameliorate cardiovascular risk factors, including hypertension and HFD-induced obesity. Dietary spermidine supplementation has been proven safe with no adverse effects reported and well-tolerated in healthy volunteers (74, 76), and older adults at risk for dementia (77). Further larger and long-term clinical investigations are needed to elucidate whether cardiovascular risk factors may be counteracted by ingesting polyamine-rich food items, polyamine-enriched plant extracts, synthetic spermidine, or by stimulating polyamine synthesis in the gut microbiome through supplementation of prebiotics or probiotics.

### Resveratrol

The polyphenol resveratrol, which is abundantly found in the skin of grapes and red wine, is one of the most extensively studied natural and *bona fide* caloric restriction mimetics. Interest in the cardiovascular health-promoting properties of resveratrol has been greatly influenced by experimental studies, demonstrating that resveratrol protects against metabolic disturbances induced by HFD and, thus, prevent early mortality in obese mice (78). The favorable effects of resveratrol on the cardiovascular system could be, at least in part, explained by its capability to promote vasodilation (79, 80), suppress atherosclerosis (81), improve glucose tolerance and insulin sensitivity (78, 82, 83), inhibit LDL oxidation (84, 85), and decrease plasma triglycerides and cholesterol accumulation (86). In addition to reported protection from the negative consequences of an obesogenic diet, such as insulin resistance (87), resveratrol has been demonstrated to exhibit anti-inflammatory effects (88, 89). The anti-inflammatory properties of resveratrol include down-regulation of genes involved in inflammatory pathways (90), as well as systemically inhibited expression of TNFα, IL-6 (90, 91), IL-1β, ICAM-1, and iNOS (91). Altogether, the anti-inflammatory activity has been postulated to explain a relatively low risk of cardiovascular disease in the French population consuming moderate amounts of resveratrol in red wine, despite high intake of saturated fats (so-called “French Paradox”) (92).

Evidence has accumulated indicating that resveratrol, both *in vivo* and at nutritionally relevant concentrations *in vitro*, can activate several interrelated signaling pathways in the cardiovascular system. Many of the beneficial cardiovascular effects of resveratrol are mediated by pathways that require SIRT1 in cardiomyocytes and endothelial cells (93, 94). Although both SIRT1 and AMPK are necessary for resveratrol-induced health promotion (87, 95), there are likely other molecular targets of resveratrol that contribute to its cardioprotective effects. Studies reported that resveratrol inhibits the nuclear factor kappa-light-chain-enhancer of activated B-cells (NF-kB) pathway (96), attenuates vascular oxidative stress (97, 98), and upregulates eNOS (99, 100), which is known to improve endothelium-dependent vasodilation through increased nitric oxide bioavailability. Importantly, SIRT1-mediated activation of autophagy is a key process in mediating many beneficial effects of resveratrol (101–103). Very recently, resveratrol was found to promote lysosomal function via endoplasmic reticulum calcium-dependent TFEB activation, which is associated with reduced intracellular lipid accumulation (104). Importantly, inhibition of mTORC1 activity and presence of Unc-51-like kinase 1 (ULK1) were shown to be required for autophagy induction by resveratrol (105). However, although resveratrol attenuates the activation of mTORC1, low dose resveratrol reportedly induces the expression of Rictor, a component of mTORC2 pathway (106). Overall, despite the large number of molecular targets that have been identified responsible for the promiscuous effects of resveratrol, more research effort is needed before definitive mechanisms can be assigned to its multifaceted cardioprotective benefits. On the basis of available evidence, it can be endorsed that resveratrol-induced cardiovascular protection is controlled by many of the pathways (e.g., NF-kB pathway) and master regulators (e.g., mTORC) involved in cellular stress resistance, redox homeostasis and cellular energetics.

Encouraging results from preclinical research have greatly increased the interest in resveratrol supplementation to mitigate cardiovascular risk factors in humans. A recent meta-analysis of 17 randomized clinical trials validated the blood pressure lowering effect of resveratrol (107). The anti-hypertensive effect of resveratrol that was consistently reproduced only in studies testing doses >300 mg/day was reported mainly in patients with diabetes mellitus type 2 likely due to its favorable effect on insulin sensitivity (108). Of note, lower systolic blood pressure is associated with metabolic changes (90). In this small randomized control trial, 30 days of resveratrol supplementation decreased intrahepatic lipid content, circulating levels of glucose and triglycerides, and inflammation markers, while it stimulated adipose tissue lipolysis in obese men. By contrast, a recent study failed to demonstrate the efficacy of resveratrol against metabolic syndrome (109). In fact, although resveratrol has been shown to modify risk factors in experimental models of obesity and cardiovascular diseases by phenocopying most of the transcriptional aspects and molecular mechanisms of caloric restriction, including the suppression of inflammatory response (91, 110), it is important to note that clinical trials mostly failed to reproduce cardiometabolic improvements likely due to low *in vivo* bioavailability of resveratrol (111). This is particularly relevant because *in vivo* evidence has been viewed increasingly important in endeavors to understand how resveratrol elicits its effects in humans and to ascertain the optimum doses and routes for mitigating cardiovascular risk factors. To this end, other small-molecule activators of SIRT1 have been developed. For instance, SRT1720 has been demonstrated to extend lifespan and improve metabolic syndrome, insulin sensitivity, and endothelial dysfunction in mice, while a related compound, SRT2104, has undergone clinical phase I and II trials, revealing only minor adverse effects (112). Interestingly, rapid metabolism of resveratrol and the composition of the gut microbiome were proposed to control the production of resveratrol metabolites, which are detected at higher levels in humans after intake than their parent compound, with similar biological effects (113). Owing to its capability in modulating the composition of the gut microbiota, resveratrol may affect central energy metabolism and modify concentrations of satiety hormones to produce anti-obesity effects. Similar to resveratrol and spermidine, fasting also induces changes to the gut microbiome and improves immune homeostasis with a sustained beneficial effect on body weight and blood pressure in hypertensive patients with metabolic syndrome (114), suggesting that caloric restriction mimetics and dietary interventions promote cardiovascular health at least in part by regulating the abundance of certain microbes in the gut (115).

### NAD^+^ Precursors

Recent years have witnessed growing interest in NAD^+^ intermediates as molecules that efficiently recapitulate the salutary effects of caloric restriction and exercise by elevating cellular NAD^+^ content, which is reduced in aging, obesity and other metabolic disorders (116). Direct supplementation of NAD^+^ precursors, in particular nicotinamide riboside (NR) and nicotinamide mononucleotide (NMN), has been shown to alleviate metabolic abnormalities by reducing body weight gain and reinstating blood glucose control in mice consuming HFD (117, 118). Along similar lines, nicotinamide (NAM, also known as vitamin B3) was found to improve glucose homeostasis associated with positive effects on liver metabolism in absence of obesity-lowering effects in aged mice fed HFD (119). Recently, we have also demonstrated that orally administered NAM to male and female ZSF1 obese rats with cardiometabolic syndrome evidently reduces hyperphagia-induced obesity (120). This effect could be partially attributed to increased energy expenditure and improved metabolic flexibility. In addition, NAM moderately lowers high arterial blood pressure, while it improves diastolic dysfunction in ZSF1 obese rats, *Dahl* salt-sensitive rats and aged mice (120). In another study, oral NMN supplementation late in life to aged mice was also found to elicit anti-aging effects on the vasculature by improving aortic stiffness in association with increased arterial SIRT1 activation and reduced vascular oxidative stress, suggesting that NMN delays arterial aging and its pathological sequelae (121).

Mechanistically, increased NAD^+^ is required for a sustained SIRT1 deacetylase activity, which regulates autophagy through deacetylation of autophagy-related proteins, such as ATG5, ATG7 and ATG8 (122). In addition, NAD^+^ can induce autophagy via AMPK (123). The NAD^+^/sirtuin pathway activates mitophagy, which was demonstrated to maintain cardiac function during HFD-induced diabetic cardiomyopathy (124). Moreover, NR supplementation was shown to activate SIRT1 and SIRT3, improve mitochondrial function and protect against HFD-induced obesity in mice (118). It is important to mention, however, that other NAD^+^-modulated processes, like inflammation and oxidative stress, which are attenuated by NAD^+^, might be involved in the cardiac and more broadly physiological effects of NAD^+^ precursors. In fact, health-promoting effects of NAM coincide with reduced inflammation, oxidative stress and adipose tissue infiltration with leukocytes (119, 120).

Ample preclinical evidence has demonstrated that strategies to increase NAD^+^ content can mitigate cardiovascular disease in various rodent models. Hence, NAD^+^ precursors are increasingly proposed as promising agents to reduce the burden of cardiometabolic diseases in humans. Niacin, which has been typically used in the form of nicotinic acid, is the most extensively studied NAD^+^ precursor in humans. The impact of niacin on lipid control and cardiovascular risk in humans was recently re-examined in a meta-analysis based on a systematic review of 119 clinical trials that included 35,760 patients (125). Collectively, this analysis revealed a marginal benefit of niacin as a monotherapy to elevate HDL-cholesterol levels, but raised doubts about the safety profile of niacin, especially in combination with statins. Despite its poor tolerability, niacin remains in use as an alternative lipid-lowering agent in statin-intolerant patients at cardiovascular risk. First reports on human trials that tested other NAD^+^ boosting strategies than niacin have only started to emerge (126), announcing an era of NAD^+^ therapeutics. Amongst these, NR and NMN are the main precursors in ongoing or lately completed clinical trials (127). In fact, a recent study in postmenopausal, overweight women with prediabetes, demonstrated that 10 weeks of NMN supplementation increases skeletal muscle insulin signaling, insulin sensitivity, and muscle remodeling (128). These beneficial metabolic effects of NMN supplementation differ from the observations reported from NR trials conducted in obese middle-age and older men and women (129–131), suggesting different biological functions of NMN and NR. Another clinical investigation showed that NR may have the potential for reducing blood pressure and aortic stiffness in healthy middle-aged and older individuals (132). Additionally, NR has been shown to exert anti-inflammatory effects not only in aged healthy individuals, but also in hospitalized patients with heart failure (129, 133). Of note, high doses of oral NAM are safe and have also been shown to reduce non-melanoma skin cancers as well as markers of cardiorenal injury (134), opening a new perspective on the previously understudied therapeutic potential of NAM. In this regard, a diet enriched in NAM and NA is associated with lower blood pressure and a reduced risk of overall and cardiac-specific mortality in humans (120).

Taken together, several challenges need to be overcome before experimental findings on rodent models of cardiovascular risk factors can be translated into clinics. Future clinical trials need to be of longer duration and include a follow-up assessment, involve large numbers of patients, and consider more appropriate conversion of drug doses from rodent studies to human trials (135). In this regard, quantification of potential long-term adverse effects will be instrumental to ensure that NAD^+^ precursor administration at higher doses is safe for the use in humans. Head-to-head studies are warranted to answer the outstanding question about the optimal NAD^+^ precursor, and determine which of the NAD^+^ precursors have superior properties, capable of eliciting a wide range of beneficial effects that may improve cardiovascular risk factors. In addition, several practical hurdles will need to be overcome, such as how to best deliver NAD^+^ precursors to achieve the optimal NAD^+^ bioavailability, and at what dose and time of the day, as NAD^+^ levels are subjected to circadian fluctuations. Future studies should also compare the effects, efficacy and outcomes of pharmacologically increased NAD^+^ levels *vs*. physiological means of raising NAD^+^ levels, such as regular physical activity and dietary interventions that are designed for older individuals with comorbidities.

### Metformin

The biguanide metformin, which originates from the French lilac, is the first-line drug used for the treatment of diabetes mellitus type 2 (136). Although best known for its glucose-lowering effects, a growing body of evidence indicates that metformin extends lifespan and healthspan (137) by mitigating age-associated conditions (138, 139), such as cancer, cognitive decline and cardiovascular diseases (140) across various species (137, 141, 142). Metformin exhibits a plethora of direct effects on the cardiovascular system. For example, it potently protects against hypertrophy in a pressure overload rat model, likely via increased AMPK and eNOS phosphorylation and higher nitric oxide production (143), leading to improved endothelial function and vasodilation (144). Metformin effectively reduces atherosclerotic plaque size in high-cholesterol diet fed rabbits by decreasing high-sensitivity C-reactive protein and inhibiting the NF-kB pathway in the vascular wall (145). In addition, metformin is capable of stabilizing atherosclerotic plaques by activating AMPK in *ApoE*-knock-out mice (146), resulting in better cardiovascular outcomes as calcification of plaques is associated with their instability and serves as a negative predictor of mortality (147, 148). Metformin attenuates inflammatory response in rabbits fed an atherogenic diet by reducing infiltration of macrophages (149), which is known to result in their differentiation to foam cells and atherosclerotic plaque formation (150). Furthermore, metformin suppresses the NLRP3 inflammasome and upregulates autophagy in mice with diabetic cardiomyopathy through the activation of AMPK and inhibition of mTORC (151, 152), both of which regulate aging-related pathways, leading to prolonged lifespan (153). Furthermore, metformin increases the expression and activity of SIRT1, while it attenuates the activation of PGC1α, a central energy metabolism regulator (154).

As most of the research endeavors focused on the glucose-lowering effect of metformin, it is not surprising that the majority of clinical trials were designed to investigate the beneficial role of metformin on diabetes mellitus type 2. However, several human studies assessed the impact of metformin monotherapy on other age-associated comorbidities as well. For example, metformin reduces pro-inflammatory cytokine levels in older diabetic patients, suggesting that metformin has the potential to attenuate age-related low-grade chronic inflammation, reduce the predisposition toward inflammation-related comorbidities, and improve survival of diabetic patients (155). In another clinical investigation, the use of metformin was assessed in the context of cardiovascular outcome in patients with diabetes mellitus type 2 and chronic kidney disease (156). The authors that analyzed data from the TREAT trial (157) demonstrated that metformin reduces the incidence of cardiovascular events as well as cardiovascular death and all-cause mortality. Importantly, metformin was found to be safe for patients with chronic kidney disease, which is in contrast with the previous assertion that metformin commonly induces lactic acidosis (158). In pubertal children with diabetes mellitus type 2 and metabolic syndrome, metformin improves various health parameters, including BMI, leptin levels, fat mass and liver fat (159). Interestingly, some of these beneficial effects were maintained after completing the 24 months of metformin treatment, suggesting that metformin is well-tolerated and has a potential long-term benefit in adolescents at risk. In the REMOVAL trial, patients with diabetes mellitus type 1 displayed lower LDL-cholesterol levels after 3 years of metformin treatment (160). Recently, a meta-analysis that included 16 studies and nearly 2 million participants revealed that metformin reduces overall cardiovascular risk, including mortality and incidence, in patients with diabetes mellitus type 2 (161). Another comprehensive meta-analysis of 260 studies described a general drop in all-cause mortality and occurrence of cardiovascular disease in diabetic patients upon metformin treatment as compared to diabetic patients receiving other medication and, interestingly, even non-diabetic subjects (139). These observations highlight that metformin could extend lifespan and healthspan by acting as a geroprotective drug. However, studies in healthy or non-diabetic populations are rare and showed conflicting results. For example, the CAMERA study failed to produce the beneficial effects of metformin on cardiovascular disease prevention in non-diabetic patients with high cardiovascular risk (162). By contrast, 6 weeks of metformin treatment reduced body weight, improved insulin secretion, lowered LDL and triglyceride levels in an elderly population exhibiting impaired glucose tolerance but no previous history of diabetes (163).

Of note, the 6-year Targeting Aging with MEtformin (TAME) clinical trial^3^, which started in 2016 as a large randomized controlled and multicenter study, including over 3,000 participants (between the ages of 65–79) without diabetes but who are at high risk for the development of chronic diseases of aging, is expected to generate highly valuable new knowledge about the impact of metformin on the primary outcome of death and major age-related chronic disease development, such as cardiovascular disease, cancer, and dementia (164).

## Future Perspectives and Concluding Remarks

Recent years have seen a growing interest in understanding how dietary interventions shape and interact with the most common cardiovascular risk factors, including hypertension, obesity, metabolic syndrome, and diabetes mellitus type 2. Substantial cardiometabolic improvements have been reported with fasting interventions such as reduction in blood pressure, body weight and fat mass, lower blood glucose, and improvement in insulin sensitivity, both in experimental and clinical studies. Although caloric restriction consistently improves several aspects of health, its application has been hampered by poor compliance and adverse side effects on bone health and immune response, especially in the elderly. To overcome these major hurdles, clinical trials on alternate-day or intermittent fasting, with higher statistical power and follow-up, are strongly needed before they can be implemented as a treatment strategy. Individuals practicing alternate-day or intermittent fasting should consider to include regular physical activity to maintain their energy expenditure. Emerging evidence indicates that the optimal cardioprotective diet is constructed around the traditional Mediterranean eating pattern.

Another interesting aspect that warrants further attention is the effect of caloric restriction mimetics or dietary interventions aimed at weight loss on the gut microbiome changes in obese patients with diabetes mellitus type 2 or metabolic syndrome. Although these interventions propose beneficial clinical outcomes, their effect on the gut microbiome is only beginning to unfold. Interestingly, a combination therapy of resveratrol and spermidine synergistically induces autophagy at doses, which do not trigger effects of the same magnitude if administered alone. At present, however, it remains elusive what is the optimal dose for any of the caloric restriction mimetics that could provide health benefits or protect humans at risk of cardiovascular disease.

Unlike the current drug development approaches that focus on individual diseases in isolation and consider specificity as a desirable outcome in disease prevention and treatment, both caloric restriction mimetics and caloric restriction exhibit a spurious mode of action, intercepting with multiple different targets (165). Such pleiotropic mode of action appears advantageous in targeting the complex process of aging as the greatest risk factor for cardiovascular diseases and associated comorbid conditions. Thus, dietary interventions should aim to maintain optimum health and prevent cardiovascular diseases by attenuating the molecular causes of biological aging directly.

Non-cell autonomous effects of caloric restriction mimetics and caloric restriction itself, such as the anti-inflammatory or immune modulatory functions, are increasingly viewed as relevant as cell autonomous mechanisms. Taking this into account, more research is needed to ascertain how different forms of fasting and caloric restriction mimetics can be the most favorable to further improve cardiometabolic markers in healthy adults and patients living with or at risk of developing cardiovascular disease. Based on the currently available data, harnessing caloric restriction mimetics or dietary interventions, such as intermittent fasting or the Mediterranean diet represent a promising preventive venue, which might reduce cardiovascular risk and the burden of cardiovascular disease.

## Author Contributions

SS conceptualized the manuscript. JV, MA, and SS contributed to the research for writing the manuscript. JV and SL-H designed the figures and table. All authors contributed to the discussion, writing, and review of the manuscript.

## Funding

This work was supported by the Austrian Science Fund–FWF (I3301) and the European Research Area Network on Cardiovascular disease (ERA-CVD, MINOTAUR) to SS. MA acknowledges funding received from the European Society of Cardiology, the Austrian Society of Cardiology (Präsidentenstipendium der ÖKG), and the Medical University of Graz (START Fund). SL-H reports funding by the Austrian Science Fund - FWF (V530) and BioTechMed-Graz (Young Researcher Groups [YRG]).

## Conflict of Interest

MA and SS are involved in a patent application related to the cardiometabolic effects of caloric restriction mimetics. The remaining authors declare that the research was conducted in the absence of any commercial or financial relationships that could be construed as a potential conflict of interest.

## Publisher's Note

All claims expressed in this article are solely those of the authors and do not necessarily represent those of their affiliated organizations, or those of the publisher, the editors and the reviewers. Any product that may be evaluated in this article, or claim that may be made by its manufacturer, is not guaranteed or endorsed by the publisher.

## Footnotes

^1^World Health Organization. Health topics - cardiovascular diseases (2019). Available online at: https://www.who.int/health-topics/cardiovascular-diseases#tab=tab_1 (accessed July 29, 2021).

^2^World Health Organization. Health topics - diabetes (2019). Available online at: https://www.who.int/health-topics/diabetes#tab=tab_1 (accessed July 29, 2021).

^3^American federation for AGING RESEARCH. The TAME Trial (2021). Available online at: https://www.afar.org/tame-trial (accessed July 29, 2021).
